# The Pupil‐Brain System at Rest: Spontaneous Pupil Fluctuations as Markers of Neuromodulatory and Network Dynamics

**DOI:** 10.1111/psyp.70338

**Published:** 2026-06-09

**Authors:** T. Liu, S. A. Kotz, A. Criscuolo, M. Schwartze

**Affiliations:** ^1^ Department of Neuropsychology & Psychopharmacology, Faculty of Psychology and Neuroscience Maastricht University Maastricht the Netherlands; ^2^ Department of Neuropsychology Max Planck Institute for Human Cognitive and Brain Sciences Leipzig Germany

**Keywords:** neuromodulation, pupil‐brain coupling, spectral coupling, spontaneous pupil fluctuations

## Abstract

Spontaneous pupil fluctuations (SPFs) during rest provide a non‐invasive, low‐cost index of central arousal dynamics, independent of cognitive task demands. These features position SPFs as promising markers for baseline neurophysiological activity in both basic and translational research. This review synthesizes current evidence on the resting‐state pupil‐brain system, focusing on its core components: central neuromodulatory circuits and large‐scale cortical networks. We first examine the relationship between SPFs and distributed elements of the ascending arousal system, including noradrenergic, cholinergic, serotonergic, and dopaminergic nuclei. We then detail SPF coupling with intrinsic functional networks—default mode, salience, and sensorimotor systems—highlighting their role in mediating transitions between internally‐ and externally‐oriented brain states. Finally, we discuss the spectral and directional properties of pupil‐brain interactions, emphasizing analytical approaches suited for assessing directionality and outlining implications for clinical and translational applications. Converging evidence from animal and human neuroimaging studies reveals robust spatiotemporal and spectral coupling between SPFs and neural activity across micro‐ and macro‐scales. These findings support a systems‐level framework in which SPFs serve as integrative markers linking subcortical neuromodulation with large‐scale cortical dynamics. We conclude that SPFs offer a sensitive window into arousal regulation and body–brain integration, with potential utility as biomarkers for neuropsychiatric conditions and altered states of consciousness.

## Impact Statement

Combining evidence from human and animal studies, this review proposes a systems‐level framework positioning spontaneous pupil fluctuations (SPFs) as integrative markers of neuromodulatory and cortical network dynamics. It highlights how SPFs bridge subcortical arousal systems and large‐scale brain organization, offering a non‐invasive approach to study intrinsic brain states and potential biomarkers for neuromodulatory dysfunction.

## 1 Introduction

Pupil size, commonly referred to as pupil diameter, has emerged as a non‐invasive, high temporal resolution marker for assessing central arousal dynamics and the activity of neuromodulatory systems (de Gee et al. 2017; Joshi and Gold 2020; Lloyd et al. 2023; Pfeffer et al. 2022). Pupil‐linked measures provide access to fluctuations in the activity of subcortical arousal systems such as the locus coeruleus‐norepinephrine (LC‐NE) pathway (Joshi et al. 2016; Murphy et al. 2014), and have been widely used to study attention, learning, and cognitive control across species (Aston‐Jones and Cohen 2005; de Gee et al. 2014; Eldar et al. 2013; Meissner et al. 2024).

Pupil research has focused on event‐related and resting‐state fluctuations. Event‐related experiments inevitably modulated cognitive demands, which may influence or phase‐reset ongoing pupil and neural activity and reflect transient, stimulus‐locked activity rather than continuous dynamics (de Gee et al. 2014; Urai et al. 2017). In contrast, resting‐state paradigms allow monitoring of endogenous, spontaneous pupil fluctuations (SPFs), which manifest as ultra‐slow oscillations (around 0.1–0.2 Hz) associated with variations in neuromodulatory tone (Aston‐Jones and Cohen 2005; Murphy et al. 2014; Reimer et al. 2016). Moreover, because pupil diameter serves as a covert physiological marker, it enables indirect tracking of neuromodulatory dynamics without requiring active task engagement (Lloyd et al. 2023; Pais‐Roldán et al. 2020; Yellin et al. 2015). This covert nature is particularly important because it enables the study of neuromodulatory dynamics in populations where active task performance is difficult or impossible, such as young children, minimally verbal individuals, or patients with cognitive decline.

The parasympathetic and sympathetic branches of the autonomic nervous system are the principal circuits controlling pupil size (Mathôt 2018; McDougal and Gamlin 2015). Parasympathetic control of the pupil is mediated via the Edinger‐Westphal nucleus and the sphincter pupillae muscle, whereas sympathetic pathways innervate the dilator pupillae muscle and promote pupil dilation. Higher level circuits, including neuromodulatory systems and cortical control regions, can further modulate and interact with these lower‐level circuits (Strauch et al. 2022). At the systems level, SPFs are temporally coupled with fluctuations in large‐scale functional networks, including antagonistic interactions between the default mode network (DMN) and task‐positive systems such as salience network (SN), attention networks, and sensorimotor networks (SMN) (Pfeffer et al. 2022; Schneider et al. 2016; Yellin et al. 2015). Spontaneous pupil dilations tend to coincide with increased activity in salience and frontoparietal regions, whereas pupil constrictions are associated with increased activity in visual and sensorimotor areas (Schneider et al. 2016). This bidirectional pattern suggests that pupil dynamics index ongoing shifts in large‐scale brain state, potentially reflecting a cognitive shift between externally‐oriented sensory‐motor processing and internally‐oriented states (e.g., self‐referential thought, memory retrieval, mind‐wandering; Menon and Uddin 2010; Seeley et al. 2007). SPFs also display frequency‐ and region‐specific associations with cortical oscillations (Pfeffer et al. 2022; Podvalny et al. 2021). These dynamic patterns reveal how the brain and pupil continuously regulate baseline arousal and energy allocation in the absence of external stimulation.

Within a broader framework of body–brain interactions (Criscuolo et al. 2025, 2022; Engelen et al. 2023; Kluger et al. 2024), SPFs can be considered alongside classical bodily rhythms such as heart rate (HR) and respiration rate (RR), which also covary with brain activity. Cardiac and respiratory cycles are well‐defined quasi‐periodic rhythms with identifiable features (e.g., R‐peaks in ECG, respiratory phases) and standardized analysis metrics (Malik 1996; Shaffer and Ginsberg 2017). In contrast, SPFs lack a dominant periodicity and instead exhibit broadband, irregular dynamics across multiple timescales (Joshi and Gold 2020; Reimer et al. 2016; Yellin et al. 2015). Recent evidence shows that even such aperiodic pupil fluctuations covary with the rhythmic cycle of respiration (Kluger et al. 2024; Schaefer et al. 2025), indicating that SPFs remain partially coupled to slower bodily rhythms despite their irregular structure. While this aperiodicity makes SPF quantification more challenging, it also provides a finer grained window into intrinsic neural state fluctuations, such as changes in arousal and neuromodulatory tone. Consequently, pupil dynamics offer a non‐invasive, temporally rich signal that complements traditional physiological body and brain rhythms.

This review is a narrative synthesis informed by a targeted literature search. Relevant studies were identified primarily through PubMed (U.S. National Library of Medicine; https://pubmed.ncbi.nlm.nih.gov/) and Web of Science (Clarivate Analytics; https://www.webofscience.com/). The literature search was conducted in May 2025. This review focused on studies investigating the relationship between pupil dynamics and spontaneous brain activity at rest, with particular emphasis on work published over the past decade. In the present review, “SPFs” refer to ongoing, time‐varying changes in pupil size that occur in the absence of explicit event‐related stimulation. The term “rest” refers to a state characterized by the relative absence of structured task demands. The operational definition of this state varied across the included studies, encompassing fixation‐based paradigms, blank‐screen viewing, pre‐task baseline conditions, and other low‐demand passive states. Search themes included pupil‐related terms such as “pupil size”, “pupil diameter”, “pupillometry”, and “spontaneous pupil fluctuations”, combined with terms related to resting‐state paradigms and brain function, including “resting state”, “task‐free”, “intrinsic activity”, “brain activity”, “spontaneous brain activity”, “neural correlates”, and “neuromodulation”.

Studies were prioritized if they directly investigated resting‐state or spontaneous pupil dynamics and their neural correlates in either humans or animals. Using these criteria, the core body of literature prioritized in this review comprised *n* = 12 human studies and *n* = 8 animal studies (key study characteristics are listed in Table 1). Some task‐based studies were also considered when they provided important mechanistic insights or translational context relevant to the interpretation of SPFs. The review is organized around four core dimensions that reflect emerging trends in pupil‐related research. First, we examine the neuromodulatory mechanisms that underlie SPFs. Second, we explore how SPFs relate to fluctuations in large‐scale cortical networks, highlighting their temporal coordination with brain dynamics. Third, we focus on the spectral characteristics of SPFs and how they couple with neural oscillations across timescales and modalities. Finally, we consider the clinical and translational relevance of the pupil‐brain system in neuropsychiatric and neurocognitive contexts. With this review, we seek to extend existing frameworks that conceptualize pupil dynamics as part of broader body–brain interactions (Criscuolo et al. 2025; Engelen et al. 2023; Joshi and Gold 2020). Specifically, we focus on resting‐state SPFs, synthesizing multimodal evidence relating intrinsic neural and SPFs dynamics in large‐scale network organization, and the potential use of SPFs for clinical applications. The review, thus, summarizes the role of SPFs in the body–brain system across levels of analysis, ranging from basic mechanisms to clinical applications. Taken together, the evidence reviewed across these different levels suggests that SPFs are best understood as reflecting complex system dynamics, and their fluctuations likely shaped by interactions among multiple neurotransmitter systems and cross‐network modulations. However, the precise contribution of these factors remains not completely understood.

**TABLE 1:** Overview of studies included in this review.

Study	Species/Sample	State/Condition	Neural measure	Duration
Murphy et al. (2014)	Human (*N* = 14)	Eyes‐open resting state (central fixation; constant luminance)	fMRI	8 min
Yellin et al. (2015)	Human (with fixation *N* = 20; without fixation *N* = 11)	Eyes‐open resting state (with or without fixation; constant luminance)	fMRI	8 min
Schneider et al. (2016)	Human (Original sample *N* = 32; Replication sample *N* = 36)	Eyes‐open resting state (central fixation; dark and light conditions in the original sample; mild sleep restriction in the original sample; light‐condition replication sample without sleep restriction)	fMRI	2 runs, 12 min each
Breeden et al. (2017)	Human (*N* = 35)	Eyes‐open resting state (central fixation; constant luminance)	fMRI	No report
DiNuzzo et al. (2019)	Human (*N* = 15)	Steady‐state attentional task (central fixation; darkness; constant luminance)	fMRI	2 runs, 5 min each
Ceh et al. (2020)	Human (*N* = 44)	Eyes‐open resting state (central fixation; darkness)	EEG	2 min
Mäki‐Marttunen (2021)	Human (*N* = 49)	Eyes‐open resting state (central fixation; constant luminance)	fMRI	10 min
Podvalny et al. (2021)	Human (*N* = 24)	Eyes‐open resting state (central fixation)	MEG	pre‐ and post‐task; 5 min each
Montefusco‐Siegmund et al. (2022)	Human (*N* = 16)	Inactive wakefulness (central fixation; darkness)	EEG	1 min
Pfeffer et al. (2022)	Human (*N* = 81)	Eyes‐open resting state (central fixation; constant illumination)	MEG	5–10 min
Lloyd et al. (2023)	Human (*N* = 72)	Eyes‐open resting state (central fixation; constant luminance)	fMRI	2 runs, 5 min each
Carro‐Domínguez et al. (2025)	Human (*N* = 17)	Natural sleep (darkness; right eye taped open)	EEG	4 h
Reimer et al. (2014)	Mouse (*N* = 38)	Quiet wakefulness	Whole‐cell recording; two‐photon calcium imaging	No report
McGinley et al. (2015)	Mouse (*N* = 5)	Quiet wakefulness (head‐fixed; constant low‐light conditions)	Whole‐cell recording; multi‐unit activity; LFP	No report
Joshi et al. (2016)	Monkey (*N* = 5)	Passive fixation (head‐fixed; fixation point; constant illumination)	Single‐unit electrophysiology; LFP	1–5 s per trial
Reimer et al. (2016)	Mouse and rat (*N* = 3 and *N* = 21)	Quiet wakefulness (head‐fixed; constant illumination)	Two‐photon calcium imaging; CNiFER imaging	No report
Yüzgeç et al. (2018)	Mouse (*N* = 7)	Natural sleep (head‐fixed; darkness)	ECoG; EMG	4 h
Sobczak et al. (2021)	Rat (*N* = 10)	Anesthetized resting state (head‐fixed)	fMRI	15 min 25 s
Joshi and Gold (2022)	Monkey (*N* = 3)	Passive fixation (head‐fixed; fixation spot; constant illumination)	Single‐unit electrophysiology	1–5 s per trial
Neyhart et al. (2024)	Mouse (*N* = 34)	Quiet wakefulness (head‐fixed; darkness)	Two‐photon imaging	No report

*Note:* Duration refers to the recording duration reported for the relevant state/condition; “No report” indicates that the duration was not explicitly reported in the original article.

Abbreviations: CNiFER, cell‐based neurotransmitter fluorescent engineered reporter; ECoG, electrocorticography; EEG, electroencephalography; EMG, electromyography; fMRI, functional magnetic resonance imaging; LFP, local field potential; MEG, magnetoencephalography; N, sample size.

## 2 SPFs and Central Neuromodulatory Systems

The ascending arousal system (AAS) is a distributed network of subcortical neuromodulatory nuclei that exerts a widespread influence on cortical excitability, behavioral responsiveness, and autonomic regulation. This system comprises the locus coeruleus (LC), basal forebrain (BF), dorsal and median raphe nuclei (DR/MR), and the ventral tegmental area/substantia nigra (VTA/SN) (Jones 2020; Saper et al. 2005). Recent work has linked these neuromodulatory structures to fluctuations in pupil size, making them key candidates for understanding the central drivers of SPFs and arousal in general.

Pupil research has focused primarily on the LC‐NE system. There is now strong evidence that task‐evoked and spontaneous pupil dilations link to LC activation (Joshi et al. 2016; Murphy et al. 2014; Privitera et al. 2020). However, a recent human resting‐state fMRI study also revealed positive correlations between SPFs and BOLD signals across multiple AAS nuclei, including LC, VTA, SN, DR, MR, and BF, with peak correlations occurring at a lag of 0–2 s (Lloyd et al. 2023). Such findings suggest that beyond the LC components, other nuclei of the AAS contribute to pupil modulation, thus demanding to extend the traditional LC‐centric perspective (Joshi and Gold 2020; Larsen and Waters 2018). In the following sections, we review the functional relevance of several key AAS subcomponents (the LC‐NE system, the cholinergic system, the serotonergic system, and the dopaminergic system), with a particular focus on evidence delineating how their dynamic co‐fluctuations are reflected in spontaneous pupil size changes.

### 2.1 Noradrenergic System

The noradrenergic system is one of the central neuromodulatory systems and has been consistently linked to pupil dynamics. Within this system, the pontine LC is the brain's principal source of norepinephrine. The LC projects diffusely to nearly the entire forebrain and plays a central role in regulating arousal, attention, and cognitive flexibility (Aston‐Jones and Cohen 2005; Sara and Bouret 2012). According to the adaptive gain theory (Aston‐Jones and Cohen 2005), the LC‐NE system operates in distinct phasic and tonic firing modes that support different behavioral states: the LC phasic mode facilitates responses to task‐relevant stimuli, while the LC tonic mode is associated with exploratory behaviors and lower attentional control (Aston‐Jones and Cohen 2005). However, emerging evidence suggests that LC firing displays more complex patterns than simple tonic and phasic modes. Recent large‐scale cross‐species meta‐analyses indicate diverse spontaneous LC firing patterns, which are further modulated by biological factors such as age, sex, and species‐specific brain states (Kelberman et al. 2025). Rather than indicating a state of diminished attentional control, the tonic mode encompasses a functional subdimension where brain states are actively optimized for sensory processing. By modulating intra‐thalamic dynamics, LC activity facilitates transitions toward more efficient information transmission (Caestecker et al. 2025; Rodenkirch et al. 2019). SPFs should consequently be conceived as more than simple markers of arousal but may rather reflect dynamic fluctuations in brain states and processing performance.

Supporting this view, converging evidence demonstrates a close functional coupling between LC activity and pupil diameter (de Gee et al. 2017; Joshi et al. 2016; Lloyd et al. 2023; Murphy et al. 2014). Most of the existing evidence linking LC activity to pupil size stems from task‐based research. Studies manipulating cognitive load, perceptual uncertainty, or decision demands have shown pupil dilation patterns consistent with hypothesized LC activation profiles, such as phasic firing in response to cognitive demand (Beatty 1982a, 1982b; Einhäuser et al. 2008; Murphy et al. 2011). Beyond task‐based studies, pharmacological manipulations further support a noradrenergic contribution to pupil control. Agents that reduce central noradrenergic activity, such as clonidine, tend to decrease pupil diameter, whereas agents that enhance noradrenergic signaling, such as yohimbine or atomoxetine, tend to increase it (Hou et al. 2005; Orlando et al. 2024; O'Callaghan et al. 2025; Phillips et al. 2000a, 2000b). Similarly, sleep studies show that pupil dynamics covary with endogenous fluctuations in arousal across wakefulness and sleep, further supporting the interpretation that SPFs reflect neuromodulatory state changes, including processes in which the LC‐NE system is likely involved (Carro‐Domínguez et al. 2025; Morad et al. 2000; Yüzgeç et al. 2018). In humans, Carro‐Domínguez et al. (2025) measured pupil size during overnight sleep and showed that pupil size continues to fluctuate across different temporal scales, with pupil size being inversely related to the occurrence of sleep spindle clusters (a marker of sleep resilience), and influencing the cortical response to auditory stimulation, most notably in delta power (a marker of restorative sleep functions).

More recent studies have extended this link to resting state activity. In monkeys, Joshi et al. (2016) showed that SPFs during passive fixation are temporally coupled with spiking activity and LFP signals in the LC. LC spikes were followed by transient pupil dilations with a peak at around 310 ms (Joshi et al. 2016). Joshi and Gold (2022) further showed that LC spiking systematically preceded the pupil dilation phase, with an estimated delay of about 270 ms from peak LC modulation to the relevant pupil change. In mice, Reimer et al. (2016) found that spontaneous pupil dilations during quiet wakefulness were preceded by activity in cortical noradrenergic axons originating from the LC, with peak cross‐correlation to pupil diameter occurring at a lag of about 1 s. Noradrenergic signals showed stronger correlations with the derivative of the pupil diameter. Megemont et al. (2022) confirmed a monotonic relationship between LC spiking and pupil dilation in awake mice, while also showing that this relationship varied substantially across neurons and sessions. Taken together, these results corroborate previous task‐based findings, showing that LC activity and pupil fluctuations are tightly coupled. LC signals consistently precede pupil dilations even in task‐free conditions, although the precise lag depends on the signal type and analytic measure used. This temporal precedence suggests that LC activity does not merely respond to transient cognitive demands but instead reflects a continuous, endogenous, and dynamic mechanism regulating arousal and cortical gain across behavioral states.

Human resting‐state fMRI studies have also confirmed the tight coupling between SPFs and LC BOLD signals. Murphy et al. (2014) used neuromelanin‐sensitive MRI to localize the LC and found that spontaneous pupil diameter changes positively correlated with LC activity at rest. This was further substantiated by Lloyd et al. (2023), who showed that pupil size and LC activation were temporally coupled within a narrow time window of 0–2 s, suggesting near‐simultaneous fluctuation patterns. This nearly synchronous coupling implies that SPFs track ongoing variations in LC activity, providing a sensitive and temporally precise index of noradrenergic state dynamics even in the absence of external stimuli.

### 2.2 Cholinergic System

In addition to the LC‐NE system, the BF‐cholinergic system also plays a key role in shaping cortical states associated with pupil dynamics. Cholinergic tone supports cortical desynchronization, enhances sensory responsiveness, and facilitates transitions between vigilance states (Larsen and Waters 2018; Lee and Dan 2012).

Recent animal studies directly linked cholinergic activity to SPFs. Reimer et al. (2016) found that cholinergic axonal activity in the cortex increases during pupil dilations. Although both cholinergic (acetylcholine, ACh) and noradrenergic signals were temporally correlated with changes in pupil size, ACh activity exhibited a slower rise and shorter temporal lead relative to the peak of pupil dilation (mean lag approximately 0.5 s), suggesting distinct temporal dynamics between these neuromodulatory inputs. The same study also found that smaller dilations seem to reflect isolated cholinergic input, while larger dilations involve concurrent activation of both cholinergic and noradrenergic systems, indicating combined neuromodulatory engagement during higher arousal events. Extending these findings, Neyhart et al. (2024) showed that SPFs co‐varied with cortical ACh dynamics. Cortical ACh levels and BF cholinergic axonal activity were found to be highly correlated with SPFs in awake, head‐fixed mice, especially during quiet wakefulness (non‐motor period). These findings suggest that subtle fluctuations in pupil size reflect state‐dependent cholinergic modulation even in the absence of overt behavior. SPFs capture a continuum of neuromodulatory control that bridges local cortical state regulation and global arousal dynamics. Extending these animal findings, human resting‐state fMRI work further suggests that SPFs are also coupled to BF activity, supporting a cholinergic contribution to the resting‐state pupil‐brain system (Lloyd et al. 2023).

Other evidence supports the functional significance of this coupling. For instance, McGinley et al. (2015) demonstrated that intermediate pupil diameters correlate with an intermediate arousal state most favorable for sensory processing and behavioral performance, characterized by reduced low‐frequency (1–10 Hz) and enhanced high‐frequency (30–80 Hz) desynchronized cortical activity. This suggests that cholinergic tone from the BF may help maintain this optimal cortical excitability. Causal links have also been shown through pharmacological manipulation. In this context, the cholinergic coupling observed in Reimer et al. (2016), Neyhart et al. (2024), and Lloyd et al. (2023) suggests that BF‐related pupil dynamics may contribute to the regulation of cortical excitability and sensory readiness across spontaneous state fluctuations. These findings suggest that SPFs not only reflect LC‐NE mediated arousal dynamics but also capture cholinergic contributions to cortical state regulation, highlighting the multiplex neuromodulatory origins of spontaneous pupil signals.

### 2.3 Serotonergic System

The serotonergic system is another key component of the AAS. It primarily consists of the dorsal raphe nucleus (DRN) and median raphe nucleus (MRN), and is involved in regulating mood, arousal, and switches between vigilance states. Serotonin (5‐HT) projections extend widely across cortical and subcortical regions, modulating electrophysiological slow‐wave activity, autonomic tone, and motivational states (Liu et al. 2020; Seo et al. 2019).

Although less frequently studied in relation to SPFs, existing animal research suggests a role for serotonergic modulation in baseline arousal as indicated by pupil size. For example, optogenetic stimulation of DRN serotonergic neurons can modulate tonic arousal levels and influence pupil diameter, particularly during quiescent or drowsy states (Cazettes et al. 2021). Some pharmacological studies have implicated serotonergic activity in pupil control. For instance, serotonin reuptake inhibitors (SSRIs) are known to cause mild pupil dilation in humans, and serotonergic agents such as LSD and psilocybin have been shown to reliably induce pupil dilation (Holze et al. 2022; Schmid et al. 2015). Although these effects are not specific to resting‐state conditions, they suggest that serotonergic tone can modulate pupil diameter indirectly.

Extending these observations to humans, Lloyd et al. (2023) further reported that SPFs covary with activity in the DRN and MRN. More broadly, recent work further indicated that serotonergic neuromodulation may interact with the LC‐NE and cholinergic systems in shaping global brain states and arousal levels (Aston‐Jones and Cohen 2005; Carter et al. 2010; Grandjean et al. 2019; Joshi et al. 2016; Reimer et al. 2016). However, evidence directly linking serotonergic fluctuations to SPFs at resting‐state remains sparse. Future studies combining resting‐state pupillometry with serotonergic pharmacological manipulations, or EEG/MEG measures of central neural dynamics may help clarify the distinct and overlapping roles of serotonin in pupil‐brain dynamics.

### 2.4 Dopaminergic System

Compared to the LC‐NE system and the cholinergic system, the role of dopamine in SPFs at rest remains less explored. Nonetheless, midbrain dopaminergic nuclei such as the VTA and SN are core components of the AAS and contribute to motivation, behavioral activation, and state regulation (Jones 2003; Szabadi 2025; Trutti et al. 2019), all of which have been linked to pupil‐linked arousal (Leong et al. 2021; Salamone and Correa 2024). Similarly, Lloyd et al. (2023) showed that SPFs are coupled with BOLD signal fluctuations in the VTA and SN, with strongest coupling observed at short lags of up to approximately 2 s. This finding is interpreted as reflecting broader AAS involvement in resting‐state pupil dynamics.

Pupil dilation has been linked to reward anticipation, effort allocation, and action invigoration, processes that are also strongly shaped by dopaminergic mechanisms (Kurniawan et al. 2021; Muhammed et al. 2016; Varazzani et al. 2015). In primates, simultaneous recordings of dopaminergic and noradrenergic neurons during reward/effort trade‐off behaviors have revealed a functional dissociation. Specifically, dopaminergic neurons primarily signal anticipated outcomes before movement begins, while noradrenergic activity tracks the actual mobilization of effort and the accompanying pupil dilation throughout the execution phase (Varazzani et al. 2015). This pattern suggests that pupil signals may not provide a direct real‐time readout of dopamine in the same way they are often discussed for LC‐related arousal, but may still capture behavioral and arousal‐state changes that are partly shaped by dopaminergic signaling.

Pharmacological and clinical observations are also consistent with dopaminergic influences on pupil function. Dopamine agonist treatment has been associated with altered static and dynamic pupillary responses in humans, and longitudinal work in Parkinson's disease suggests that chronic dopaminergic medication increases baseline pupil diameter in a dose‐dependent manner (Ava et al. 2022; Bartošová et al. 2025). Although these findings support the view that pupil size is sensitive to dopaminergic state, most evidence comes from task‐based designs. While informative, there is so far little evidence for the contribution of dopamine in SPFs at rest.

### 2.5 Interactions Among Neuromodulatory Systems

Although often considered separately, neuromodulatory systems should not be viewed as acting independently (Avery and Krichmar 2017; Briand et al. 2007), SPFs are likely to emerge from coordinated activity across interacting components of the AAS (Collins et al. 2023; Lloyd et al. 2023; Munn et al. 2021). Extensive anatomical and functional evidence indicates that these systems are highly interconnected, and that they jointly regulate arousal, vigilance, and cortical states (Lee and Dan 2012; Maness et al. 2022).

Interactions between the LC‐NE and BF‐cholinergic systems are especially relevant. Both systems promote wakefulness and cortical activation (Collins et al. 2023; Maness et al. 2022), but do so based on some unique temporal and functional features. LC activity is more strongly associated with prolonged alertness and broad arousal regulation, whereas BF‐cholinergic systems are particularly important in attentional effort and sensory recruitment (Maness et al. 2022). Together with evidence that noradrenergic and cholinergic signals show different temporal relationships with pupil dynamics (Reimer et al. 2016), these findings support the view that SPFs may reflect temporally multiplex contributions from both systems rather than a unitary arousal signal (Collins et al. 2023).

A similar argument applies to serotonergic influences. The serotonergic control of pupil size may not operate independently of the LC‐NE system (Larsen and Waters 2018). In mice, phasic activation of dorsal raphe serotonergic neurons increases pupil size and pupil‐linked arousal; these effects may be partly mediated by interactions with LC‐related circuits (Cazettes et al. 2021; Maheu et al. 2025; Yu et al. 2004). This is consistent with the view that SPFs reflect the combined state of interacting arousal systems, rather than the output of any one neuromodulatory system alone (Avery and Krichmar 2017).

Dopaminergic contributions are likely to be embedded within this broader network architecture. Dopamine and norepinephrine have often been described as overlapping neuromodulatory systems with partially dissociable but strongly interacting roles in vigilance, action, reward, and behavioral state regulation (El Mansari et al. 2010; Ranjbar‐Slamloo and Fazlali 2020). Accordingly, pupil‐linked changes associated with motivation, effort, or behavioral activation may reflect not only dopaminergic or noradrenergic processes in isolation but also their coordinated influence on behavioral and cortical states (Varazzani et al. 2015).

**FIGURE 1:**
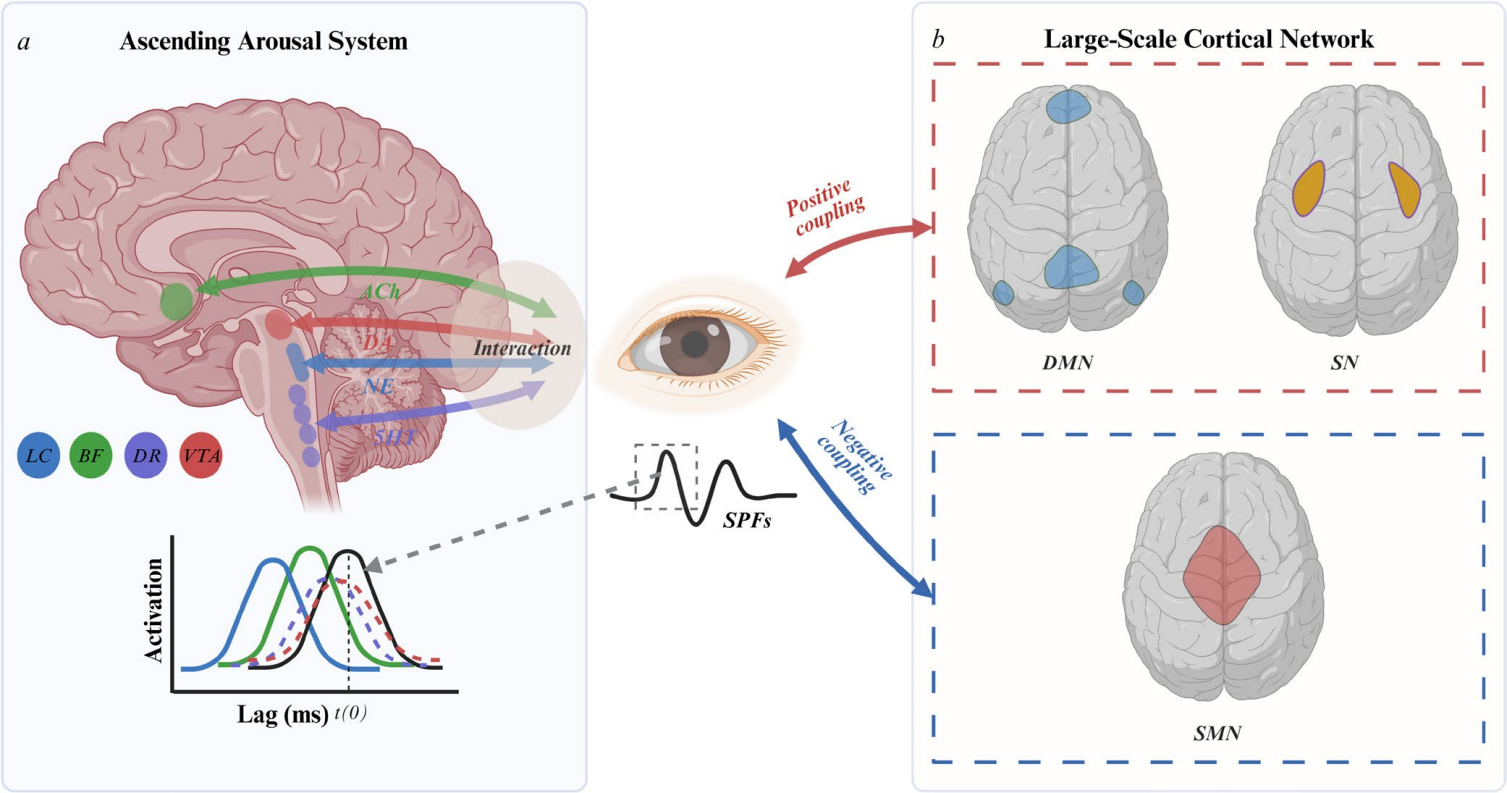
Schematic overview of the resting‐state pupil‐brain system. The figure summarizes system‐level links between SPFs, distributed neuromodulatory systems, and large‐scale cortical network dynamics. (a) Schematic illustration of neuromodulatory contributions to SPFs. The ascending arousal includes the locus coeruleus (LC; blue), basal forebrain (BF; green), dorsal raphe nucleus (DR; purple), and ventral tegmental area (VTA; red), which project widely to the cortex and interact to regulate arousal. The lower inset schematically summarizes relative temporal relationships between neural activity and pupil fluctuations. Animal studies show that LC spiking precedes transient pupil dilations on the order of a few hundred milliseconds, whereas cortical noradrenergic and cholinergic axonal signals precede SPFs on the order of approximately 1 and 0.5 s, respectively (Joshi et al. 2016; Reimer et al. 2016). The dashed DR/5‐HT and VTA/DA traces represent broader serotonergic and dopaminergic contributions, based on less temporally resolved human resting‐state fMRI evidence showing pupil‐related covariation in these nuclei within a broader lag window (Lloyd et al. 2023). The inset therefore provides a qualitative rather than quantitative comparison of relative temporal profiles. (b) Schematic illustration of large‐scale cortical networks coupling associated with SPFs. Across resting‐state studies, SPFs have been associated with positive coupling in higher‐order cortical networks, including the default mode network (DMN; broadly including medial prefrontal cortex, posterior cingulate cortex/precuneus, and inferior parietal regions) and salience network (SN; including anterior insula and dorsal anterior cingulate cortex), and negative coupling in the sensorimotor network (SMN; including primary sensorimotor cortex and supplementary motor regions). Positive coupling refers to an association between larger pupil size or dilation and relatively increased activity in the corresponding network, whereas negative coupling refers to an association with relatively reduced activity. This schematic highlights that pupil‐linked arousal fluctuations may reflect large‐scale network dynamics rather than the activity of any single cortical network.

Taken together, the pupil‐brain system at rest is influenced by interacting neuromodulatory systems operating across partially overlapping spatial and temporal scales. This view supports a multiplex account of SPFs, in which LC‐NE, cholinergic, serotonergic, and dopaminergic systems make distinct yet interacting contributions with different temporal profiles (see Figure 1a). It will therefore be important to further characterize how interacting components of the AAS are dynamically coordinated across neural and mental states, and how these joint patterns are reflected in SPFs.

## 3 SPFs and Large‐Scale Cortical Network Fluctuations

SPFs are not exclusively linked to the neuromodulatory activity of subcortical nuclei (Murphy et al. 2014) but also co‐vary with the dynamics of large‐scale cortical network dynamics. In resting state, SPFs have been associated with spontaneous fluctuations in functional brain networks such as DMN, SN, and SMN. These functional networks are known to exhibit intrinsic BOLD and electrophysiological variability in the absence of external stimuli (Fox et al. 2005) and are tightly modulated by arousal‐related neuromodulatory inputs (Shine et al. 2016). As schematically illustrated in Figure 1b, these networks show distinct patterns of coupling with SPFs during rest. In this section, we summarize the current evidence linking SPFs to fluctuations in large‐scale cortical networks during rest.

### 3.1 Default Mode Network (DMN)

The DMN is a key brain system that shows high levels of spontaneous activity during rest and is consistently implicated in self‐referential processing, episodic memory retrieval, and self‐generated thought (Andrews‐Hanna et al. 2010; Buckner et al. 2008; Menon 2023). Its core nodes include the medial prefrontal cortex (mPFC), posterior cingulate cortex (PCC), precuneus, inferior parietal lobule (IPL), and medial temporal structures such as the parahippocampal gyrus (Buckner et al. 2008; Raichle et al. 2001). These regions exhibit highly synchronized spontaneous activity during rest but also show decreased activation during externally‐directed tasks (Shulman et al. 1997). Their activity conversely increases during internally focused cognitive tasks such as autobiographical memory recall or mind‐wandering (Andrews‐Hanna et al. 2014).

When attention is directed toward externally‐oriented tasks with relatively high cognitive demands, the DMN typically shows relative suppression or deactivation. This pattern is a central feature of the classical functional framework of the DMN (Buckner et al. 2008; Menon 2023; Shulman et al. 1997). At the same time, many task‐based studies have shown that pupil dilation often accompanies increased cognitive effort, salience processing, or heightened arousal. This makes it intuitively plausible to expect that pupil‐linked increases in arousal may correspond to reduced DMN activity. However, subsequent resting‐state fMRI investigations have challenged such a simplistic dichotomy, suggesting that SPFs may also play a role in spontaneous arousal regulation during rest, rather than merely signaling activation or suppression of DMN. A study by Yellin et al. (2015) reported robust positive correlations between slow pupil size changes and BOLD signals in key DMN hubs, including the PCC, mPFC, precuneus, and IPL. Using a lag‐based correlation analysis, which quantifies the temporal delay between two fluctuating signals, the authors demonstrated that BOLD signals in these regions peaked approximately 2–3 s after changes in pupil diameter. Consistent with this general pattern, Breeden et al. (2017) also reported positive coupling between SPFs and several DMN‐related posterior regions, particularly the PCC, precuneus, and IPL. Schneider et al. (2016) did not observe a straightforward positive association between pupil size per se and canonical DMN activity; their analysis of spontaneous pupil dilations revealed positive correlations in several DMN‐related regions. Mäki‐Marttunen (2021) further showed that pupil‐indexed arousal was associated with altered DMN network organization. Specifically, higher pupil size bins were linked to greater DMN integration and lower segregation across rest and task states.

These seemingly divergent findings are better understood within a state‐dependent framework. Internally‐ and externally‐focused network states reflect distinct attentional modes, with the former involving DMN‐mediated internal cognition (e.g., memory, self‐reflection) and the latter engaging task‐positive networks that support attention to external stimuli and goal‐directed behavior (Buckner et al. 2008; Christoff et al. 2016; Fox et al. 2005; Shine et al. 2016; Smallwood and Schooler 2015). During externally‐oriented task contexts, pupil dilations are more typically associated with heightened arousal and task‐related reconfiguration toward more integrated, task‐relevant network states, often accompanied by DMN suppression and task‐positive network engagement (Buckner et al. 2008; Fox et al. 2005; He et al. 2023; Murphy et al. 2014; Shine et al. 2016). By contrast, during rest, SPFs may reflect ongoing regulation of internal arousal and intrinsic coordination among large‐scale networks, rather than simply indicating DMN suppression. These findings highlight that the same pupil dynamic marker can correlate with either increased or decreased DMN activity, depending on the cognitive context. Nobre and Gresch (2025) proposed that the brain dynamically shifts between external oriented sensory processing and internal oriented representations, with dorsal frontoparietal systems and DMN‐related regions respectively contributing to these modes. They further suggest that neuromodulatory systems mediate such shifts, particularly the LC‐NE pathway, which regulates network dynamics according to contextual demands. Such a theoretical framework aligns with the network integration model proposed by Shine et al. (2016), suggesting that arousal‐related neuromodulation, as indexed in part by pupil size, plays a central role in dynamically reconfiguring the brain between internally‐ and externally‐focused network states.

Together, these findings suggest that the relationship between SPFs and DMN activity is more complex than a simple binary modulation between DMN‐active and DMN‐suppressed states. Rather than being uniformly antagonistic, context demands appear to nonlinearly modulate pupil‐linked arousal fluctuations and may reflect transitions within the DMN itself, or between the DMN and task‐positive networks.

### 3.2 Salience Network (SN)

The SN comprises the anterior insula (AI) and dorsal anterior cingulate cortex (dACC) (Seeley et al. 2007). It is thought to support the detection of behaviorally relevant internal and external events and to contribute to the dynamic switching between the DMN and task‐positive control networks (Menon and Uddin 2010; Seeley et al. 2007). Recent meta‐analytic and modeling work suggests that arousal‐related systems, including cingulo‐insular circuitry, may contribute to the modulation of large‐scale brain network activity, providing a broader framework for understanding the SN's involvement in pupil‐linked arousal and brain‐state fluctuations (Sabat et al. 2025). In resting‐state fMRI studies, Murphy et al. (2014) and Breeden et al. (2017) both reported positive correlations between pupil size and BOLD activity in the dACC and AI. This association suggests that transient arousal fluctuations are reflected in the engagement of salience‐related regions. Further evidence comes from Schneider et al. (2016), who examined the first temporal derivative of the pupil signal to capture phasic dilations (reflecting rapid arousal‐related changes during rest) and found increased activation in SN regions. These phasic dilations might index brief, spontaneous shifts in internal state that prepare the brain for potential environmental engagement (Schneider et al. 2016). However, the same study did not find significant SN activation (e.g., in dACC or AI) in relation to pupil size, nor did Yellin et al. (2015) in their resting‐state analysis. This discrepancy may partly reflect differences in the pupil features examined, as Schneider et al. (2016) focused on transient pupil dilations rather than pupil size per se.

The SN may provide a cortical route through which neuromodulatory arousal signals reshape large‐scale network organization. In mice, Zerbi et al. (2019) showed that chemogenetic activation of the LC rapidly increased brain‐wide functional connectivity, particularly in salience‐related networks, together with pupil dilation. LC‐linked arousal can influence not only local activity but also broader network organization. Furthermore, one primate study has shown that LC firing is closely related to changes in coordinated activity patterns in the ACC (Joshi and Gold 2022). The authors concluded that ongoing LC activity was associated with reduced pairwise correlations in the ACC during passive fixation, whereas transient LC responses evoked by external events were associated with enhanced ACC coordination. Moreover, by analyzing neural activity relative to the ongoing pupil phase, the authors showed that ACC signals displayed phase‐related changes that preceded those of LC spiking. Altogether, these findings suggest that salience‐related cortical coordination may not simply reflect LC‐driven arousal but may instead form part of a more dynamic interaction loop linking LC activity with pupil fluctuations.

Similar conclusions have also been drawn in task‐based contexts. When processing salient events, pupil dilation was linked to SN‐mediated resetting and switching of functional brain networks, suggesting that the coupling of SN and pupil is not limited to passive rest but extends to conditions in which the brain must rapidly reallocate resources between internal and external demands (He et al. 2023; Menon and Uddin 2010). Under acute stress, the SN may further support a hypervigilant state that facilitates threat detection (van Marle et al. 2009; van Oort et al. 2017). Taken together, these findings suggest that, even at rest, SPFs are not only associated with activity in salience‐related regions but also reflect brief fluctuations in arousal level and large‐scale neural network dynamics.

### 3.3 Sensorimotor Network (SMN)

The sensorimotor system includes the precentral gyrus (primary motor cortex), postcentral gyrus (primary somatosensory cortex), paracentral lobule, and supplementary motor area (SMA) (Woolsey et al. 1979). These structures are involved in motor planning, somatosensory processing, and bodily self‐awareness (Blanke et al. 2015; Haggard 2017; Johansson and Flanagan 2009; Nachev et al. 2008). In most resting‐state fMRI studies, SPFs exhibit significant negative correlations with BOLD activity in core regions of the SMN (Breeden et al. 2017; Lloyd et al. 2023; Mayeli et al. 2020; Schneider et al. 2016; Yellin et al. 2015). These regions consistently show reduced BOLD activity during periods of increased pupil size. Schneider et al. (2016) further reported that phasic increases in pupil diameter also relate to transient suppression of sensorimotor activity and concurrent activation of salience network regions, suggesting that arousal changes involve a reallocation of neural activity between sensorimotor and salience systems.

This pupil‐linked suppression of SMN activity often co‐occurs with increased DMN activity (Breeden et al. 2017; Yellin et al. 2015). This pattern suggests that arousal fluctuations, as indexed by pupil size, may dynamically shift the brain between DMN‐dominant introspective and sensorimotor‐dominant states (DiNuzzo et al. 2019; Lloyd et al. 2023; Mayeli et al. 2020). Such an antagonistic relationship is consistent with evidence for a functional antagonism between DMN and SMN (DiNuzzo et al. 2019; Shine et al. 2016). More generally, these findings support the view that resting‐state brain activity may reflect spontaneous fluctuations between internally‐ and externally‐oriented modes (Deco et al. 2013; Raichle 2015), even in the absence of external input (Breeden et al. 2017; Schneider et al. 2016; Yellin et al. 2015). Accordingly, the pupil emerges as a valuable proxy for intrinsic brain regulation, indexing not only sensorimotor systems at rest but also the tuning of attentional systems between the external and internal environment (Joshi and Gold 2020; Lloyd et al. 2023; Murphy et al. 2014; Nobre and Gresch 2025; Reimer et al. 2016; Shine et al. 2016).

In sum, evidence suggests that SPFs co‐vary with dynamic activity in large‐scale cortical networks. Their coordinated modulation in conjunction with SPFs reflects global, system‐level transitions in brain states, capturing the dynamic interplay between interoceptive and exteroceptive processing as well as shifts between internally‐ and externally‐oriented cognitive modes.

## 4 Spectral Structure of SPFs and Their Coupling With Brain Dynamics

Beyond showing co‐fluctuations with neuromodulatory systems and large‐scale cortical networks, the SPFs signal exhibits distinct spectral properties that can be isolated through frequency‐decomposition methods. These fluctuations span a wide range of timescales, from rapid, sub‐second phasic dilations to slow drifts lasting tens of seconds, which together enable SPFs to capture both transient and sustained changes in cognitive and neural processing modes (Nowak et al. 2008; Podvalny et al. 2021; Villalobos‐Castaldi et al. 2016). During prolonged low arousal states, the pupil can exhibit slow, rhythmic oscillations known as “hippus”, typically occurring at around 0.2 Hz (Bouma and Baghuis 1971; Mathôt 2018). In the following subsections, we summarize the different forms of pupil‐brain spectral coupling reported in the literature, discussing their directionality in the context of resting‐state neural dynamics.

### 4.1 Spectral Coupling Between SPFs and Brain Dynamics

SPFs during rest not only exhibit structured temporal coupling with brain state dynamics, but also show systematic coupling with neural oscillatory activity across multiple frequency bands. This spectral dimension affords a frequency‐resolved window into how peripheral arousal signals dynamically and bidirectionally interact with intrinsic neural rhythms. Critically, capturing this coupling requires electrophysiological methods with sufficient temporal resolution, such as scalp electroencephalography (EEG), magnetoencephalography (MEG), electrocorticography (ECoG), and depth electrode recordings (e.g., stereo‐EEG or local field potentials [LFP] in animals) to resolve directionality and avoid conflation of distinct physiological processes. In rodents, pupil dilation is closely associated with cortical activation states, characterized by reductions in low‐frequency (delta‐theta, < 10 Hz) power and increases in high‐frequency (gamma) LFP power, consistent with desynchronization and heightened arousal (Reimer et al. 2014). In contrast, pupil constriction is associated with enhanced low‐frequency synchronized states, reflecting reduced cortical arousal (Reimer et al. 2014). These phenomena have been consistently observed across visual, somatosensory, and auditory cortices, verified via both LFP recordings (Vinck et al. 2015) and membrane potential changes (McGinley et al. 2015; Reimer et al. 2014).

Human EEG and MEG studies mirror these findings. Resting‐state EEG revealed a positive correlation between SPFs and alpha‐band (8.5–12.5 Hz) power in posterior cortices (particularly the parietal‐occipital cortex) (Ceh et al. 2020; Montefusco‐Siegmund et al. 2022). Montefusco‐Siegmund et al. (2022) observed dynamic phase coupling between the two signals, with alpha power peaking 310 ms before pupil dilation peaks and reaching its minimum 348 ms before pupil constriction troughs. Human MEG data corroborate these effects at the network level, showing that pupil‐linked desynchronization is expressed across large‐scale cortical networks, including the visual, dorsal‐attention, frontoparietal, and default‐mode networks (Podvalny et al. 2021).

Pfeffer et al. (2022) additionally highlighted frequency‐ and region‐specific patterns in SPF‐cortical spectral coupling, showing that low‐frequency (2–4 Hz) power is negatively correlated with pupil size across the widespread cortical regions, while high‐frequency (64–128 Hz) power is negatively correlated in posterior visual cortices but positively correlated in prefrontal regions. In contrast to the low‐ and high‐frequency patterns described above, the relationship between pupil size and activity in the alpha/beta band (8–32 Hz) shows a complex spatial pattern, with positive correlations in occipital visual cortex and negative correlations in sensorimotor cortices, suggesting a trade‐off between cortical regions. The alpha/beta suppression in the SMN converges with fMRI evidence of SMN deactivation (see Section 3.3), providing cross‐modal support for this effect and adding spectral evidence to functional interpretations of pupil‐linked shifts between sensorimotor and introspective network states.

Importantly, the relationship between pupil size and alpha/beta power is considered to be nonlinear. Hence, several resting‐state studies showed an inverted‐U shape, whereby alpha/beta power peaks at intermediate pupil sizes and decreases at lower and higher arousal levels (Pfeffer et al. 2022; Podvalny et al. 2021). This nonlinear spectral pattern, particularly evident in parietal, occipital, and temporal regions, aligns with the Yerkes‐Dodson law (Sadaghiani and Kleinschmidt 2016; van Kempen et al. 2019), according to which intermediate arousal may be associated with more efficient processing. However, this functional interpretation remains tentative. Despite some evidence from task‐based studies combining pupillometry and EEG, current evidence more directly supports the presence of a nonlinear spectral association between SPFs and neural oscillations than its behavioral significance. Behavioral studies that combine pupillometry and EEG have primarily focused on task‐related (or event‐locked) pupil and neural fluctuations in isolation (e.g., Hong et al. 2014; van Kempen et al. 2019), ultimately leaving out spectral coupling dynamics. We hope future work will more directly test the link between nonlinear pupil‐brain spectral and behavioral performance. For an overview of studies examining frequency‐specific coupling between SPFs and cortical activity across modalities, see Table 2.

**TABLE 2:** Spectral coupling between SPFs and cortical activity across studies.

Type of study	Study (Method)	Frequency band	Correlation with pupil size	Primary regions involved	Analysis type
Human	Pfeffer et al. (2022) (MEG)	2–4 Hz (delta)	Negative	Widespread cortex	Linear
		8–32 Hz (alpha/beta)	Positive (occipital); Negative (sensorimotor)	Visual cortex (+), SMN (−)	Nonlinear (inverted‐U, parietal/occipital/temporal)
		64–128 Hz (gamma)	Positive (prefrontal); Negative (visual)	PFC (+), Visual cortex (−)	Linear
Human	Podvalny et al. (2021) (MEG)	1–4 Hz (delta)	Negative	Visual cortex, DAN, DMN	Linear
		20–80 Hz (beta/gamma)	Positive	Visual cortex, DAN, DMN	Linear
Human	Ceh et al. (2020) (EEG)	8.5–12.5 Hz (alpha)	Positive	Parietal‐occipital cortex	Linear
Human	Montefusco‐Siegmund et al. (2022) (EEG)	8–13 Hz (alpha)	Positive (pupil size and high‐freq pupil components)	Posterior cortex	Phase/timing (alpha leads pupil ~310 ms)
Rodent	Reimer et al. (2014) (LFP)	< 10 Hz (delta‐theta)	Negative	Visual, auditory, somatosensory cortices	Linear
		> 30 Hz (gamma)	Positive	Visual, auditory, somatosensory cortices	Linear
Rodent	McGinley et al. (2015) (LFP + Vm)	< 10 Hz	Negative	Auditory cortex	Nonlinear (inverted‐U, Vm and behavior)
		> 20 Hz	Positive	Auditory cortex	Nonlinear (inverted‐U)

Abbreviations: DMN, default mode network; EEG, electroencephalography; LFP, local field potential; MEG, magnetoencephalography; PFC, prefrontal cortex; SMN, sensorimotor network; Vm, membrane potential.

### 4.2 Directionality Between SPFs and Brain Dynamics

Although temporal and spectral coupling between SPFs and brain dynamics is readily observable, more information about the directionality and the mechanisms underlying this coupling is necessary for a better understanding of how SPFs reflect ongoing brain state dynamics. Accordingly, a range of methods has been applied to go beyond simple correlation.

Lag‐based correlation analyses have been used to examine whether fluctuations in SPFs precede or follow changes in BOLD signals across specific networks. Yellin et al. (2015) showed that pupil size changes precede DMN activity, consistent with slow ascending neuromodulatory influences and hemodynamic delay. Granger causality and related autoregressive models provide a statistical framework to infer the time course of pupil‐brain interactions across neural networks. Using Granger causality analysis, Yellin et al. (2015) reported that activity in the posterior inferior parietal lobule (pIPL) predicted subsequent pupil size fluctuations, while in turn, pupil size predicted activity in early visual areas (EVA). These findings indicate distinct temporal dependencies that may reflect top‐down influences from higher‐order parietal regions on pupil‐linked arousal, as well as apparent bottom‐up effects in early visual cortices that could partly arise from increased visual input when the pupil enlarges. Such analyses thus aid the characterization of bottom‐up and top‐down dynamics across the visual hierarchy.

Electrophysiological methods provide finer temporal resolution. MEG studies using cross‐correlation and time‐resolved phase‐based metrics have provided evidence for rapid top‐down cortical influences on SPFs. Podvalny et al. (2021), for example, found that spontaneous cortical gamma activity preceded pupil dilation by around 400 ms and highlighted spatially specific modulation patterns, such as differential coupling of pupil fluctuations with default mode versus visual/sensorimotor networks (Breeden et al. 2017; Yellin et al. 2015).

Pharmacological interventions offer a more direct way to probe neuromodulatory contributions by manipulating transmitter systems. Gelbard‐Sagiv et al. (2018) showed that altering noradrenergic tone changed baseline pupil size, perceptual sensitivity, and late visual cortical responses. Pfeffer et al. (2021) contrasted catecholaminergic and cholinergic enhancement, reporting that NE elevation increased baseline pupil size and high‐frequency activity, whereas ACh elevation was linked to slower pupil responses and alpha/beta modulation. Although these observations were drawn from task‐based rather than resting‐state settings, they show that neuromodulator manipulations can influence both pupil dynamics and cortical activity.

Another promising approach comes from pupil‐based biofeedback paradigms. A recent study showed that individuals can volitionally modulate their pupil diameter using real‐time biofeedback, leading to systematic changes in LC activity and other brainstem arousal structures, as well as cardiovascular and behavioral responses (Meissner et al. 2024). These findings indicate that pupil fluctuations are not merely passive markers of arousal but may be integrated into active control loops that modulate body–brain dynamics. However, existing evidence utilizing bio−/neurofeedback paradigms shows a modulatory effect on brain‐behavior interplay rather than a simple one‐to‐one causal relation. Changes in the observed signal (e.g., pupil size) might be an epiphenomenon of a cascade of neurobiological processes that lead to behavioral changes, rather than a causal mechanism (Kvamme et al. 2022). Computational approaches offer a complementary route. For example, recent work has emphasized the methodological importance of modeling pupillometric data as continuous time series rather than reducing them to a few summary features, particularly because pupil signals exhibit complex temporal structure and autocorrelation (van Rij et al. 2019). At the systems level, Sobczak et al. (2021) developed a decoding framework which demonstrates that pupil‐fMRI coupling depends on the underlying network state. Such approaches provide novel tools to capture the state‐dependency of SPF‐brain relations and to test mechanistic hypotheses by comparing simulated and observed fluctuations. Together, these approaches offer complementary tools for characterizing the temporal organization and mechanistic basis of SPF‐brain coupling.

## 5 Clinical and Translational Implications of the Pupil‐Brain System

As peripheral readouts of neuromodulatory activity, SPFs provide dynamic information about brain arousal levels, attentional allocation, and cortical state modulation in the resting state. This makes them particularly attractive for clinical and translational research, especially in populations for whom task performance is difficult, unreliable, or confounded by cognitive impairment (Joshi and Gold 2020; Mathôt 2018). In clinical research, SPFs are typically interpreted together with multiple pupil‐based measures, such as resting pupil diameter, phasic pupillary responses, and pupillary light reflex (PLR). Together, these indices can provide complementary information about tonic arousal state, reactive autonomic modulation, and the functional integrity of the underlying neuromodulatory systems (Aminihajibashi et al. 2020; Mathôt 2018; Samuels and Szabadi 2008; Steinhauer et al. 2022). Many neurodevelopmental, neuropsychiatric, and neurodegenerative disorders involve disturbances in arousal regulation and autonomic control (Isaac et al. 2024; Orlando et al. 2023; Ross and Van Bockstaele 2021; Samuels and Szabadi 2008), processes that are closely linked to LC‐NE, cholinergic, and broader AAS function (Munn et al. 2021; Samuels and Szabadi 2008).

### 5.1 Autism Spectrum Disorder (ASD)

ASD has been described on the basis of differences in social‐communication behavior and restricted or repetitive behaviors, but growing evidence suggests that altered arousal and autonomic regulation also form part of its broader phenotype (Arora et al. 2021; de Vries et al. 2021). Some theoretical accounts have suggested that physiological arousal is already atypical during rest and correlates with core features of autism (Arora et al. 2021; DesLauriers and Carlson 1969; Hutt et al. 1964). Accordingly, resting‐state pupillometry may provide a reliable non‐invasive window into the pupil‐brain system in ASD.

Resting‐state and passive‐viewing studies consistently report enlarged resting pupil diameters in children with ASD, suggesting elevated tonic arousal or impaired modulation (DiCriscio and Troiani 2017, 2021; Keehn et al. 2021; Kim et al. 2022). Kim et al. (2022) further showed a pattern of increased tonic but reduced phasic LC‐NE related activity in ASD, indicating that heightened baseline arousal may coexist with attenuated adaptive responsivity. Zhao et al. (2022) found exaggerated responses to repetitive stimuli and blunted responses to novel ones in ASD. These pupillary indicators also correlate with the severity of clinical symptoms (Zhao et al. 2022). Beyond group‐level differences, pupillometric measures have also shown promise for individual‐level ASD classification. DiCriscio and Troiani (2021) showed that resting pupil diameter and pupil dilation amplitude, together with sex and IQ, classified 86.3% of participants as having an ASD diagnosis in a logistic regression model. Artoni et al. (2019) trained a convolutional neural network on SPF patterns and achieved 97% classification accuracy in ASD mouse models, and further showed through transfer learning that the same approach could detect neurodevelopmental disorder signatures in human patients.

### 5.2 Attention‐Deficit/Hyperactivity Disorder (ADHD)

ADHD is increasingly understood as a disorder that involves abnormalities in attention and arousal regulation, rather than attentional dysfunction alone. Although the exact neurobiological mechanisms of ADHD remain to be fully clarified, existing evidence points to differences in neural activity within the central nervous system, particularly in the LC (Konrad et al. 2006; Kumano et al. 2022). In addition, dysfunction in serotonergic, noradrenergic, and dopaminergic pathways has also been implicated in its pathophysiology (Cai et al. 2021; Faraone et al. 2025; Hoogman et al. 2017; MacDonald et al. 2024; Yacoub et al. 2025). Consequently, pupil size offers a promising opportunity to explore neuromodulatory dysfunction in ADHD.

Shirama et al. (2020) reported larger tonic pupil size and reduced phasic dilation during an auditory continuous performance task in adults with ADHD, indicating disrupted arousal gain control. Resting‐state work further showed that ADHD was associated with larger pupil size (Nobukawa et al. 2021; Turkoglu et al. 2025) and lower temporal complexity of SPFs (Nobukawa et al. 2021), suggesting that dysregulation extends beyond task performance into intrinsic arousal dynamics. Turkoglu et al. (2025) also found that mean pupil dilation velocity was slower in ADHD, pointing to altered autonomic regulation, including heightened sympathetic drive and impaired dynamic parasympathetic modulation. Emerging evidence has also pointed out differences in PLR metrics in ADHD, although the direction and magnitude of effects can vary across paradigms, ages, and symptom profiles (Duque‐Chica et al. 2025; Hamrakova et al. 2020). Wainstein et al. (2017) found that children with ADHD showed reduced pupil diameter, and that this difference normalized when the same children were on methylphenidate medication (a norepinephrine‐dopamine reuptake inhibitor), suggesting potential utility in treatment monitoring. At the individual classification level, Das and Khanna (2021) developed a machine learning framework that combines spectral and temporal dynamic features of pupil‐size fluctuations into an objective biomarker for automated ADHD detection, achieving robust classification performance.

### 5.3 Alzheimer's Disease (AD)

AD is a disorder of progressive memory loss and cognitive decline, with early disruption of neuromodulatory and arousal‐regulatory systems (Zhang et al. 2024). Beyond the classical finding of an accumulation of amyloid‐β and tau, converging evidence indicates that the LC is among the earliest sites of tau pathology and undergoes substantial degeneration over the course of AD, with important consequences for noradrenergic regulation of attention, cortical state, and autonomic control (Beardmore et al. 2021; Chen et al. 2022; Jacobs et al. 2021). Cholinergic dysfunction is also a hallmark of AD and is highly relevant to pupil regulation, given the role of parasympathetic pathways in pupillary constriction (Frost et al. 2017; Hussain et al. 2023; Wu et al. 2020).

Converging evidence documents abnormalities in PLR dynamics in AD, including reduced constriction amplitude, slower constriction, delayed redilation, and broader alterations in quantitative light reflex pupillometry (qLRP; an objective pupillometric assessment of light‐evoked pupil responses) measures (Chougule et al. 2019; David et al. 2025; Fotiou et al. 2007, 2009; Gramkow et al. 2024; Prettyman et al. 1997). More recent studies have moved beyond simple reflex measures and suggest that arousal‐related pupil dynamics are altered more broadly in AD. Using simultaneous resting‐state EEG and pupillometry, David et al. (2025) found that reduced peak alpha frequency (i.e., cortical slowing) and blunted phasic pupil responses were associated with reduced LC integrity and cognitive performance, with tonic pupil‐EEG covariation at rest highlighting the role of SPFs in baseline arousal in AD. Complementing this finding, recent clinical work has linked resting pupillary diameter and light‐evoked pupillary change to prognosis and cognitive decline in AD cohorts (Gramkow et al. 2025). Taken together, emerging multimodal evidence indicates that both tonic and phasic pupil measures are altered in AD.

From a translational perspective, pupil‐based measures have shown promising clinical utility in AD. A recent qLRP study reported diagnostic models with moderate discriminatory ability for distinguishing AD from healthy controls and other forms of dementia (Gramkow et al. 2024). Moreover, longitudinal work further suggests that smaller resting pupil diameter and related qLRP‐derived changes may predict subsequent clinical progression and cognitive decline in early AD (Gramkow et al. 2025). Overall, current evidence supports the view that pupil‐based indices may serve as accessible markers of neuromodulatory dysfunction and disease progression in AD.

### 5.4 Parkinson's Disease (PD)

PD is a progressive neurodegenerative disorder characterized by both motor and non‐motor signs and symptoms. PD involves abnormalities in multiple neuromodulatory systems, including dopaminergic pathways, the LC‐NE system, and cholinergic circuits relevant to autonomic regulation (Bohnen et al. 2022; Costa et al. 2023; Palma and Kaufmann 2018; Sun et al. 2023). LC dysfunction in neurodegenerative disease may itself be stage dependent. Weinshenker (2018) proposed a stage‐dependent model of LC dysfunction in which LC abnormalities in neurodegenerative diseases such as AD and PD may evolve during disease progression. Potentially from early dysfunction, and in some stages even hyperactivity, to later marked neuronal loss and noradrenergic deficiency (Weinshenker 2018). This also suggests that disease stage should be considered an important contextual factor when examining the relationship between neurodegenerative disease and SPFs, because different LC functional states may correspond to distinct pupil features and patterns of abnormality.

Multiple studies have reported changed PLR dynamics in PD, including reduced constriction velocity, lower maximum constriction acceleration, and broader evidence of autonomic imbalance, even in patients without overt autonomic symptoms (Fotiou et al. 2009; Giza et al. 2011; Tsitsi et al. 2023; You et al. 2021). These characteristics may vary with disease stage and specific clinical phenotypes, with evidence that freezing of gait is associated with greater impairment in PLR measures and that constriction velocity declines with PD progression (Alhassan et al. 2022; You et al. 2021). Evidence on SPFs at rest in PD remains limited (Dawidziuk et al. 2025). Under relatively stable viewing conditions, Tsitsi et al. (2021) revealed that baseline pupil size and fixation stability reliably distinguish PD patients from controls. Jain et al. (2011) examined pupillary unrest in darkness, a resting measure of spontaneous pupil variability reflecting fluctuations in autonomic tone, and found associations with arousal symptoms and motor severity in PD.

### 5.5 Integrative and Translational Significance of SPFs

Across the exemplary neurodevelopmental and neurodegenerative conditions included here, pupil‐based measures emerge as sensitive clinical markers of arousal dysregulation, alterations in autonomic control and neuromodulatory functions. Although ASD, ADHD, AD, and PD differ in etiology and clinical manifestation, converging evidence suggests that abnormal pupil dynamics may index shared dimensions of dysregulation that cut across conventional diagnostic categories, including altered arousal dynamics across both rapid phasic and slower tonic timescales, reduced autonomic flexibility, and instability of internal state regulation. From this perspective, SPFs are of particular interest because they may provide a more continuous window on ongoing intrinsic arousal dynamics than reflexive or task‐based pupil responses alone. However, much of the existing clinical literature relies on baseline pupil diameter, PLR, task‐evoked dilation, or pupillary unrest, rather than directly characterizing SPFs at rest and the dynamic coupling with brain activity. Current findings should therefore be taken as supporting the broader relevance of pupil‐based measures in clinical and translational research, thus motivating more clinical research on SPFs and brain‐pupil crosstalk at rest.

Differently from task‐evoked pupillary responses, SPFs can be assessed in resting‐state or passive paradigms, thus enabling their use in populations with limited task engagement, such as young children, minimally verbal individuals, or patients with cognitive decline. Moreover, SPFs provide a system‐level readout of central neuromodulatory tone, especially within LC‐NE and cholinergic pathways. From a clinical perspective, such measures could support finer patient stratification, earlier detection of neuromodulatory dysfunctions, and more individualized monitoring of treatment response or disease progression. From a translational perspective, they may help connect mechanistic models of arousal regulation and pupil‐brain coupling with new clinical applications fostered by innovations in the digital health market. They may include digital tools for passive assessment, longitudinal and remote patient monitoring, and personalized biofeedback protocols. However, substantial challenges remain, e.g., their integration into routine clinical practice requires standardized protocols for data collection and analysis.

Notably, the potential relevance of pupil‐based indices likely extends beyond the selective clinical examples discussed in the present review. Some neuropsychiatric conditions characterized by dysregulated arousal and neuromodulatory function have also begun to explore pupil measures as candidate biomarkers. For example, in post‐traumatic stress disorder, larger pupil responses to sudden sounds have been observed alongside heightened LC activity. Other work has linked increased LC activation to hyperarousal symptoms in this population (McCall et al. 2024; Naegeli et al. 2018). Related findings in anxiety and depressive disorders further suggest that pupil measures may capture alterations in threat‐ and reward‐related arousal dynamics (Castellotti et al. 2025; Schneider et al. 2020). Thus, although most of the existing literature still focuses on reflexive or task‐evoked pupil measures, these findings nevertheless support the broader possibility that the SPF‐informed pupil‐brain framework may also be relevant to a wider range of neuropsychiatric conditions.

## 6 Discussion

SPFs reflect the combined activity of multiple neuromodulatory systems within the AAS, including noradrenergic, cholinergic, serotonergic, and dopaminergic pathways. While the LC‐NE system is a primary driver, recent studies show that the basal forebrain and raphe nuclei also contribute to pupil‐linked arousal signals (Lloyd et al. 2023; Reimer et al. 2016). These systems operate at different timescales and show distinct temporal coupling with pupil dynamics. This multiplex origin supports a system‐level view of pupil control. Rather than reflecting a single source, SPFs emerge from the integrated activity of subcortical neuromodulators that also regulate cortical states. Within the body–brain system, pupil size serves as a noninvasive readout of internal state transitions shaped by the interaction of neuromodulatory circuits. State‐dependent fMRI evidence shows that pupil‐related brain patterns can vary across trials and may involve different neuromodulatory centers, rather than reflecting a single invariant pathway (Sobczak et al. 2021).

At rest, SPFs reflect not only neuromodulatory activity but also ongoing changes in large‐scale cortical networks. Across studies, SPFs have been shown to co‐fluctuate with intrinsic network dynamics, particularly within DMN, SN, SMN systems (Breeden et al. 2017; Lloyd et al. 2023; Yellin et al. 2015). These networks support different aspects of internal regulation, and their coordination mirrors shifts in global brain states. Within the pupil‐brain system, these findings suggest that pupil dynamics index transitions in cortical network organization that also occur independently of external input. The pupil thus serves as a peripheral signal that tracks large‐scale reconfigurations of brain activity, offering insight into how internal arousal and cortical state are jointly regulated in the resting brain. This supports the view of SPFs as a bridge between neuromodulatory drive and cortical network expression, spanning micro‐ to mesoscopic levels of analysis.

SPFs show structured spectral patterns that reflect dynamic coupling within the pupil‐brain system. Fluctuations in pupil size co‐vary with frequency‐specific cortical activity, including decreases in low‐frequency power and increases in high‐frequency power during dilation or larger pupil size, consistent with cortical desynchronization and elevated arousal, although these relationships are not uniform across all frequency bands and regions (Pfeffer et al. 2022; Podvalny et al. 2021; Reimer et al. 2014). These effects vary across regions and frequency bands, revealing a complex but systematic relationship between peripheral arousal and cortical state. Together, spectral and network evidence positions SPFs as a dynamic trace of how neural systems coordinate across time. Rather than treating noradrenergic, cholinergic, serotonergic, and dopaminergic systems as fully separable, an important next step is to understand how these systems dynamically interact to shape spectral organization and pupil‐linked brain states in humans.

Beyond spectral coupling, directionality analyses show that SPFs can both follow and precede brain signals at different timescales. Fast cortical activity can predict pupil changes within hundreds of milliseconds, while slower neuromodulatory influences may drive pupil‐brain co‐fluctuations over seconds (Pfeffer et al. 2022; Podvalny et al. 2021; Yellin et al. 2015). SPFs may not simply mirror arousal levels but rather reflect the bidirectional dynamics between cortical hierarchies and neuromodulatory systems. On the one hand, activity in regions such as the parietal cortex can predict subsequent SPFs, consistent with top‐down influences on arousal control. On the other hand, SPFs can modulate early processes in visual cortices, indicating a bottom‐up channel through which peripheral arousal feeds back into sensory processing. SPFs provide a potential access point to study how top‐down and bottom‐up interactions are coordinated across the visual hierarchy, complementing their role as global arousal markers, which provide the possibility of inferring underlying mechanisms of body–brain coupling using accessible peripheral signals.

From a translational perspective, the pupil‐brain system offers promising value as a non‐invasive index of neuromodulatory function. Clinical populations exhibit alterations in pupil dynamics compared to healthy controls, although the specific measures studied range from tonic diameter and spontaneous fluctuations to phasic or light‐evoked responses. Children with ASD often show enlarged tonic pupil size and enhanced low‐frequency SPF power, suggestive of elevated baseline arousal or impaired modulation, while ADHD is associated with larger tonic size and reduced phasic reactivity, reflecting disrupted arousal gain control. In neurodegenerative conditions, AD and PD patients frequently exhibit blunted or slowed pupil responses and altered resting‐state variability, consistent with LC‐NE and cholinergic dysfunction. Although these alterations do not provide diagnostic specificity, they underscore the potential of SPFs as highly accessible, non‐invasive biomarkers for patient stratification, symptom tracking, and treatment monitoring. Importantly, because SPFs can be measured during rest or passive viewing, they provide a particularly useful tool when task compliance is low, such as in young children, minimally verbal individuals, or patients with cognitive decline. Moreover, SPFs spectral features correlate with behavioral, neurophysiological, and structural markers across conditions, suggesting their value as sensitive and mechanistically meaningful biomarkers. As SPFs integrate signals from central (e.g., LC‐NE, cholinergic systems) and peripheral autonomic control systems, they offer a lens through which the dynamic coupling between brain states and physiology can be mapped. With continued methodological and computational advancements, SPFs may contribute to stratified psychiatry and neurology by enabling early detection, individualized monitoring, and mechanism‐informed intervention strategies. However, we must acknowledge that this metric remains in its developmental phase. Future work should focus on validating SPF‐based indices across larger databases, establishing standardized analytic pipelines, and examining their predictive value in longitudinal and interventional settings.

A particularly intriguing aspect is that the relationship between SPFs and brain activity is not strictly linear, but may vary across arousal stages. Across species and modalities, resting‐state studies have reported inverted‐U associations between pupil size and alpha‐ or beta‐band activity, with intermediate pupil sizes linked to distinct spectral profiles relative to lower and higher arousal states (McGinley et al. 2015; Pfeffer et al. 2022; Podvalny et al. 2021). Such findings resonate with the classic Yerkes‐Dodson law, implying that both hypo‐ and hyper‐aroused states may impair cortical information flow, whereas intermediate states promote efficient integration and adaptive behavior. This pattern shows a potential common principle through which neuromodulatory systems jointly regulate cortical excitability and behavioral flexibility. Such a principle may also underlie the integrated dynamics of the pupil‐brain system. Although existing evidence remains limited to a relatively small number of studies, further systematic characterization of this non‐linearity across modalities and populations could elucidate how the pupil‐brain system dynamically modulates neural processing to achieve optimal performance.

We note that “resting‐state” in the current review does not constitute a homogeneous condition. Although most studies are grouped under rest or other task‐free states, their specific operationalizations differed, including the illumination setting, whether continuous fixation was required, and the participant's vigilance or drowsiness level. These factors may influence the expression of SPFs and their coupling with brain activity. Accordingly, variability in network and spectral findings should not be interpreted simply as inconsistency across studies. This may rather reflect state‐dependent differences in how the pupil‐brain system is expressed across related low‐demand conditions. Studies that have directly examined such differences suggest that heterogeneity across resting‐like conditions may not fundamentally alter the overall pattern of pupil‐brain coupling, but can affect its magnitude or regional expression. Yellin et al. (2015) reported broadly similar pupil‐BOLD topographies with and without fixation, although coupling in parts of the parietal DMN was reduced in the no‐fixation condition. Similarly, Schneider et al. (2016) found that, despite some differences in baseline pupil measures and region‐specific effects, including thalamic involvement, the main pupil‐linked neural patterns were largely comparable across dark and light fixation‐rest conditions and in a nonsleep‐restricted replication sample. These findings suggest that fixation, illumination, and arousal state may shape the expression of pupil‐brain coupling without necessarily changing its overall pattern. Accordingly, resting‐state SPFs should not be viewed as arising from a single, pure, and fully uniform resting condition. They are better understood as dynamic phenomena that emerge across a range of related but not identical low‐demand states. Within these states, pupil‐brain coupling appears to show a certain degree of stability, but its magnitude, regional distribution, and network emphasis may still vary as a function of sensory context, behavioral constraint, and vigilance or arousal level. Heterogeneity in the review does not weaken the value of SPFs as a window into brain‐state fluctuations. Instead, it highlights the importance of defining and classifying experimental states more carefully when interpreting findings across studies.

Taken together, we support the view of SPFs as part of a broader body–brain system, in which fluctuations in pupil size reflect the convergence of neuromodulatory drive and cortical network dynamics. Establishing such a pupil‐brain framework is important for several reasons. On the one hand, it moves the field beyond treating pupil size as a unidimensional physiological marker toward recognizing it as a dynamic interface between neural and bodily systems. This better encourages the integration of pupillometry with multimodal recordings (EEG, MEG, fMRI, autonomic signals and/or wearables), enabling the development of more effective analytical methods. On the other hand, the pupil‐brain system supports the use of pupil signals for individualized monitoring of neuromodulatory dysfunction across disorders, and provides a foundation for using SPFs as a bridge between basic neuroscience and clinical applications.

## 7 Future Direction and Conclusion

As a temporally structured signal coordinated centrally, SPFs have emerged as a promising marker of intrinsic brain states dynamics. Within the broader framework of the pupil‐brain system, SPFs reflect not only local neuromodulatory activity but also large‐scale network reconfiguration and peripheral‐central integration. Despite growing research interest and empirical support, key conceptual and methodological challenges remain.

First, recent advances in multimodal approaches, such as simultaneous EEG/MEG and pupillometry (Pfeffer et al. 2022; Podvalny et al. 2021), together with improved imaging and localizing small brainstem structures (Liu et al. 2017; Lloyd et al. 2023), provide more access to the spatiotemporal architecture of the pupil‐brain system. In animal models, circuit‐level techniques such as optogenetics and fiber photometry offer complementary opportunities to probe the neural mechanisms underlying these dynamics (Cazettes et al. 2021; Grimm et al. 2024; Reimer et al. 2016). However, there are also many challenges to integrating signals across modalities. The aperiodic and nonstationary nature of SPFs requires more careful preprocessing and the use of multiscale or nonlinear methods to capture their dynamic complexity. Differences in timing and signal sensitivity across methods make interpretation difficult. In particular, it is not easy to align fast pupil changes with slower brain signals such as fMRI‐based BOLD activity or network‐level fluctuations. Addressing this issue will require better methods for aligning signals across time and standardized preprocessing pipelines, which can improve comparability and reproducibility of results across studies and laboratories.

Second, questions of causality remain largely unresolved. While time‐lagged correlations and directional modeling (e.g., Granger causality) have provided first findings (Yellin et al. 2015), future work may benefit from integrating computational methods such as generative modeling, dynamic causal modeling (Friston et al. 2019), and neural mass models that explicitly simulate pupil‐linked arousal fluctuations. Clarifying causal links is essential for establishing SPFs as a mechanistically grounded biomarker rather than merely correlational proxy.

Third, although an increasing number of studies links SPFs to brain dynamics (e.g., Podvalny et al. 2021; Reimer et al. 2014) and bodily signals such as HR and RR (e.g., Kluger et al. 2024; Melnychuk et al. 2021; Schaefer et al. 2025), integrative frameworks that conceptualize SPFs as part of the body–brain system are still missing. Most studies still treat pupil‐brain and pupil‐body associations in isolation. Future work should therefore aim to characterize how SPFs co‐vary with both central and peripheral signals over time, potentially revealing cross‐system principles of embodied arousal regulation. Such efforts will also support the development of unified, multimodal models that capture state transitions across neural, autonomic, and behavioral domains.

Fourth, an important unresolved question concerns how to meaningfully characterize and interpret intra‐ and inter‐individual variability in pupil control and pupil‐brain‐behavior coupling. Individuals differ in their ability to adaptively modulate arousal to optimize sensory and cognitive processing (Aminihajibashi et al. 2019; Maier and Grueschow 2021; Robison et al. 2022). Understanding the sources and functional implications of this variability may provide a critical step toward personalized models of arousal regulation.

Fifth, as summarized above, SPFs show strong potential for capturing dynamic fluctuations between interoceptive and exteroceptive attention, reflecting how the brain flexibly shifts between internally‐ and externally‐oriented states. However, current evidence is still limited and heterogeneous, mainly based on correlational findings that do not yet establish the mechanistic basis of these state transitions. Future studies should systematically investigate how SPFs relate to dynamic shifts between interoceptive and exteroceptive modes of attention. Further work should also clarify the underlying neuromodulatory and network mechanisms that link pupil dynamics to the functional balance between self‐referential and sensory processing networks.

Finally, from a translational standpoint, if individuals vary in their ability to adaptively modulate arousal levels to optimize information processing, it becomes essential to ask whether targeted intervention or training could enhance such capacity. For instance, pupil‐based biofeedback training has recently been shown to enable voluntary modulation of pupil size and LC activity (Meissner et al. 2024). Future work should examine whether similar training approaches can strengthen adaptive arousal control and improve cognitive or affective functioning in clinical populations. Establishing such interventions would bridge mechanistic and clinical research, underscoring the translational potential of the pupil‐brain system in therapeutic contexts.

As reviewed here, converging evidence from animal and human studies demonstrates that SPFs reflect the integrated activity of multiple neuromodulatory systems, co‐vary with cortical oscillations and intrinsic networks, and capture dynamic transitions between internally‐ and externally‐oriented brain states. Their accessibility and high temporal resolution position SPFs as a promising tool for bridging basic systems neuroscience with clinical applications.

## Author Contributions

**A. Criscuolo:** conceptualization, writing – review and editing, supervision, project administration. **M. Schwartze:** conceptualization, writing – review and editing, supervision, project administration. **T. Liu:** conceptualization, investigation, writing – original draft, writing – review and editing. **S. A. Kotz:** conceptualization, writing – review and editing, supervision, project administration.

## Funding

Liu, T was supported by the China Scholarship Council (CSC) [Grant No. 202408330098]. Criscuolo, A was supported by the Dutch Research Council (https://www.nwo.nl/projecten/hnuxr80789).

## Ethics Statement

This article is a review of previously published studies and did not involve human participants or animal subjects.

## Conflicts of Interest

The authors declare no conflicts of interest.

## Data Availability

The authors have nothing to report.
